# Two decades of ART: improving on success through further research

**DOI:** 10.1590/S1678-77572009000700020

**Published:** 2009

**Authors:** Christopher J. HOLMGREN, Márcia Cançado FIGUEREDO

**Affiliations:** 1 BDS, FDSRCS, PhD, Department of Global Oral Health, College of Dental Sciences, Radboud University Nijmegen Medical Centre, The Netherlands.; 2 DDS. MSc, PhD, Department of Pediatric Dentistry, Faculty of Dentistry, Federal University of Rio Grande do Sul, Brazil.

**Keywords:** Atraumatic Restorative Treatment (ART), Developing countries, Dental caries, Health services research, Dental education, Cost effectiveness

## Abstract

Since the introduction of the Atraumatic Restorative Treatment (ART) approach over twenty years ago, more than 190 research publications have appeared. The last research agenda defining research priorities for ART was published in 1999. The objective of the present work was to review existing research in the context of future research priorities for ART. Material and Methods: An internet survey was conducted amongst those who had published on ART or were known to be working on the ART approach, to solicit their views as to areas of future ART research. Three broad categories were defined, namely: 1. Basic and laboratory research; 2. Clinical research, and, 3. Community, Public Health, Health Services Research. Results: A 31% response rate was achieved. The study identified a number of new areas of research as well as areas where additional research is required. These are expressed as recommendations for future ART research. Conclusions: The ART approach is based on a robust, reliable and ever-growing evidence base concerning its clinical applications which indicates that it is a reliable and quality treatment approach. In common with all other oral health care procedures, targeted applied research is required to improve the oral health care offered.

## INTRODUCTION

The famous quotation of Albert Einstein that “If we knew what it was we were doing, it would not be called research, would it?”^25^ holds as true for nuclear physics as it does to oral health and dentistry. In spite of the explosion of dental research over recent decades, the sad fact is that the everyday practice of dentistry has not made the quantum leap to enable effective and affordable oral health care to be brought to the vast majority of the over 6.8 billion people that now inhabit our planet.

Since the mid-1980's, when Frencken pioneered Atraumatic Restorative Treatment (ART)^20^, the approach has been subjected to extensive scientific research and evaluation. The highly promising early results of a community field trial of ART in Thailand^24^, linked with the increasing realisation of a need for dental caries care to move to more minimal intervention techniques^12^^,^^13^, led to a symposium being organized to review the scientific rationale for certain minimal intervention techniques, including ART, and to propose an agenda for future research in this field. This symposium was held during the 73^rd^ General Session of the International Association of Dental Research in Singapore in 1995^55^. Following the symposium, the organizers and speakers met to define a preliminary agenda for research on minimal intervention techniques for caries, including ART^30^. Here, five broad areas for research on minimal intervention techniques for caries were identified, namely: their clinical evaluation, caries control, the development of suitable dental materials, behavioral aspects and educational perspectives of the approach. All the areas defined for minimal intervention also applied to the ART approach, ART being part of minimal intervention. Nonetheless, a research agenda specifically for ART was also defined but at this point in time was limited to an evaluation of its clinical effectiveness.

The symposium and the publication of its proceedings stimulated a number of groups around the world to pursue further research into minimal intervention techniques for caries, including ART, so that three years later, in 1998, a further symposium was organised entitled “The State of ART (Atraumatic Restorative Treatment) - a scientific perspective”. This was held as part of the 76^th^ General Session of the International Association of Dental Research in Nice, France^56^. At this symposium, Holmgren and Frencken^28^ (1999) reviewed recent research and developments with respect to ART in the context of the 1995 research agenda^30^ and outlined future areas for research and development.

Since the 1998 IADR symposium on ART^56^, there have been several international symposia devoted specifically to ART, as interest in the approach has grown almost exponentially. Those involved in oral health, from a multitude of countries, have realized the huge potential that such an approach can offer to help combat what has been termed by Edelstein^15^ (2006) as “the global pandemic of dental caries”. However, none of the symposia have been devoted specifically to ART research and thus the ART Symposium “Two decades of ART – Success through Research” held during the 3^rd^ Latin American Regional Meeting of the IADR, on Isla de Margarita, Venezuela in November 2009 provided a timely opportunity to take stock of what we have learnt about ART through research over the past two decades and identify what future direction ART research should take.

Frencken, et al.^24^ (1994) published the results of the first ART research in 1994. Since then, numerous researchers from many countries around the world have undertaken research concerning ART. Tasked with identifying areas of further ART research the authors considered it relevant and useful to solicit the views of those who have or are currently undertaking research on ART, those who have published on the subject and those that have worked with ART and were known to the authors. A survey was therefore conducted to solicit their views as to areas of future ART research.

## MATERIAL AND METHODS

To identify those who have published papers on ART, an electronic search of the digital archive of biomedical and life sciences journal literature Pubmed was undertaken in late October 2009 using the term “Atraumatic Restorative Treatment”. This search term alone was used since Mickenautsch, et al.^47^ (2009) found that the terms “ART”, “ART approach”, and “ART technique” were not sufficiently specific to select publications relating to Atraumatic Restorative Treatment. It was however realised that such a search strategy might not identify all publications that might be applicable to ART, such as related developments in the dental materials field, or those that were published in languages other than English. This Pubmed search identified a total of 176 publications dating from 1977. Six of these publications, published prior to 1994, were unrelated to the ART approach and therefore excluded.

In the abstract of publications in the Pubmed database it is becoming common practice for the e-mail address of the principal author to be provided. This was the case for 75 publications, giving a total of 66 authors to contact. Personal contacts of people who have worked on ART, known to the authors of this paper, were added to the list, totalling 76 people to contact.

A standard letter was sent to the collected e-mail addresses. The letter explained why they had been contacted and that the purpose of the exercise was to identify areas for future research on ART. It was suggested that they could propose future research, divided into three broad categories, namely: 1. Basic and laboratory research; 2. Clinical research, and, 3. Community, Public Health, Health Services Research. It was also explained that it was not obligatory to respond to all three areas of research since the person contacted might only have expertise in one of the areas of research. A reasonable deadline was also given for replies.

Of the 76 persons who were sent an e-mail, the addresses used were found to be incorrect in 29 cases since the e-mail was returned by the internet service provider. In such cases the internet Google^®^ search engine was used with the author's name to try to identify a new contact address. eventually, this resulted in a total of 66 e-mails being successfully sent. One week after the given deadline a total of 21 responses had been received representing a 31% response rate.

The responses from this internet survey were compiled for a presentation given during the symposium “Two Decades of ART – Success through Research” mentioned above. Discussions held subsequent to this symposium added several other important themes for future ART research.

Given below are areas for future ART research proposed, the justifications for the research, and specific recommendations. These are divided into the same categories as defined in the internet survey i.e. Basic and laboratory research, Clinical Research, and, Community, Public Health and Health Services Research. It is inevitable that there is some overlap between the different categories since for instance clinical research might be supported in part by a parallel laboratory investigation and vice versa.

## BASIC / LABORATORY RESEARCH

### Research to better understand the effects of ART on the dentine / pulp complex

The effect of glass ionomer as used in the ART approach on residual carious dentine has been examined by Smales, et al.^60^ (2005) in primary teeth and in permanent teeth by Ngo, et al.^51^ (2006). Both studies report penetration of the fluorine and strontium ions into the dentine which is consistent with a remineralization process. The relative effects of the antimicrobial properties of the cavity conditioner and the GIC, as against lesion starvation from sealing the cavity, on remineralisation, is not known. Furthermore, the long term effects of placing an ART restoration on residual carious dentine are unknown.

While it is not the intention to routinely leave significant amounts of infected dentine when placing an ART restoration, sometimes this is the case to avoid a pulpal exposure (see later). In such cases little is known about the effects of this on the dentine/pulp complex. Traditionally this has been examined by extracting the tooth for histological examination of the pulp. Here Kidd^34^ (2004) considers that there is a need for a method of monitoring pulpal pathology *in vivo*.

Recommendation: There is a need for further research to understand the effects of ART restorations on the dentine/pulp complex over time, relating to different levels of removal of carious dentine.

### Research to improve dental materials used for ART

Part of the recommendations for future research and development in the preliminary research agenda for minimal intervention techniques for caries, including ART^55^, concerned the need for improved dental materials^30^. This was answered in part by the development of Fuji IX^®^ (GC Dental), a high-strength glass ionomer specially developed for ART. Other manufacturers closely followed suit with similar materials such as Ketac Molar, Ketac Molar Easymix (3M ESPE) and Chemflex (Dentsply). The effectiveness of a number of these have been validated in clinical trials.

While glass ionomer cement used for ART has inherent antimicrobial properties^10^,^59^, some researchers have attempted to enhance this effect by the use of antimicrobials such as chlorhexidine^6^,^23^, or by the addition of antibiotics^68^. While all the studies have reported that these modified glass ionomers have enhanced antimicrobial action, a danger being that the physical properties of the material might be compromised^61^. For the moment the clinical outcomes of ART restorations using these modified glass ionomer materials have not been studied and thus there is a need to clinically justify the addition of antimicrobials to glass ionomer.

The objective of instrumentation with hand-instruments, as used in the ART approach, is to remove the soft, heavily infected and unremineralisable “infected dentine” leaving behind the harder, minimally infected and remineralisable “affected dentine”, thereby conserving sound tooth structure. Studies by Palma-Dibbs, et al.^53^ (2003) and Czarnecka, et al.^9^ (2007) suggest that the bond strengths of glass ionomer to affected dentine can be less than that to sound dentine. Bond strength is important when restoring cavities with little or no natural retention and therefore attempts should be made to develop systems which specifically improve the bond strength of glass ionomer to affected dentine.

On a more practical issue, the working and setting time of glass ionomers is often optimised for room temperatures which are usually of the order of 20-23°C. At higher temperatures, such as those that might well be encountered in outreach situations, the working time can be significantly decreased. This can make it difficult to pack a cavity and related fissures before the material becomes too hard to use the press finger technique. Clinical experience shows that this can sometimes lead to “high” restorations, which require substantial shaping, particularly with inexperienced operators.

Another potential complication of high temperatures is a reduced shelf life of the material. For countries where high temperatures are encountered, materials which are less sensitive to temperature need to be developed.

Recommendation: Research should continue to develop improve materials for ART which have antibacterial properties, enhanced bond strength to affected dentine and extended working time and shelf life under less than optimal conditions.

## CLINICAL RESEARCH

### Research on the individual clinical steps involved producing an ART restoration

The clinical step-by-step procedures required to produce an ART restoration have been described in detail by Frencken and Holmgren^21^ (1999). Both in this publication and during ART training courses the strict adherence to these step-by-step procedures is emphasised with the objective of obtaining reliable clinical outcomes. However, each step in a clinical procedure takes time and uses material, both of which complicate the procedure and have cost implications. While the ART step-by-step procedure is largely based on an understanding of the carious process, knowledge of the properties of the filling material (glass ionomer) and sound common sense, the necessity of some steps might be re-examined and perhaps others proposed. Here, any modifications to the standard ART step-by-step procedures should be assessed in terms of true clinical outcomes and any gains that might be accrued in terms of savings in time and materials.

In terms of the steps which might be examined or further examined are:

· the need for sharp excavators for cavity cleaning;
· other cavity cleaning approaches such as chemo-mechanical;
· the value of pre-treatment of the cavity, e.g. cavity “sterilisation”^16^^,^^18^, the use of silver fluoride^36^;
· the effect of consistency of glass ionomer^14^;
· the effect of different packing techniques;
· the need to apply a varnish or petroleum jelly to protect the restoration^52^.

Recommendation: Research should be undertaken to examine the individual clinical steps of the ART approach to determine if each step is obligatory to produce reliable clinical outcomes.

### Research on the need to remove all carious dentine and the management of deep caries lesions

In the ART approach the term “cavity cleaning” instead of “cavity preparation” is used to distinguish between the traditional mechanistic approach (cavity preparation) and a biological approach (cavity cleaning). Here, an understanding of the caries process and the extent of the caries lesion determines the size and shape of the final cavity. Thus, with this approach there cannot be a pre-conceived cavity design^21^.

As mentioned above, the intention of cavity cleaning as used with the ART approach is to remove the soft, heavily infected and unremineralisable “infected dentine”, except in deep caries lesions where there is a risk of pulpal exposure. For such cases soft dentine is deliberately left behind and the cavity filled and sealed with a sealant restoration. In this context Kidd^34^ (2004) has asked the question “how clean must a cavity be before restoration?”. In her review of this subject she concludes that even this question might be irrelevant since there is little evidence that infected dentine must be removed prior to sealing the tooth with a restoration. A Cochrane review has reported a similar finding^58^. This has implications both for minimally invasive approaches such as ART as well as for the management of deep caries lesions. The question thus turns full circle, since if it is true that infected dentine does not need to be removed for biological reasons, then the only reason to remove it, either in part or in total, would be for mechanical reasons; namely, to assist with the retention of the restoration. Here, Mertz-Fairhurst, et al.^43^ (1998), showed that it was possible to maintain very minimally prepared sealed restorations over dentinal lesions for a period of 10 years. The findings from this study need confirmation and it is exciting to learn that a multicenter randomized controlled clinical trial is underway to evaluate the effectiveness of an alternative treatment for deep caries lesions in Brazil^40^, where, in one group, carious dentine will be partially removed and a restoration placed in one session, while stepwise excavation^5^ will be used in the other group. Since in this study only amalgam or composite resin will be used, there is a need to undertake a similar form of evaluation with glass ionomer, as is used with the ART approach.

Recommendation: Further research is needed to clarify the effects of partial and no removal of “infected” dentine on clinical outcomes in terms of restoration survival and pulpal health. Partial removal should include comparisons of infected dentine removal only at the enamel-dentine junction, as against removal here and towards the pulpal floor of the lesion.

### Research on cavity size, shape and location

In order to achieve the most reliable results from the ART approach, careful selection of cases is essential. Here, factors such as cavity size, its shape and location might play an important role in predicting restoration survival. early studies^38^ showed that smaller single-surface ART restorations have a higher survival rate than larger restorations. Kemoli and van Amerongen^32^ (2009) have also studied the effect of proximal cavity size in primary molars on survival outcomes. There is however a need to undertake further work in this important area using a standardised and widely accepted method of classifying cavities, to enable this information to be easily applied to daily clinical practice. Mickenautsch and Grossman^45^ (2006) propose that the use of the classification system of Mount and Hume^49^ (1997) could be useful in this respect.

Recommendation: Further research should be undertaken to clarify the role of cavity size, shape and location on survival outcomes using a standardised and clinically applicable method of classification of cavities.

### Research on ART in multi-surface cavities

The growing number of clinical and community studies investigating the survival of ART restorations and sealants has permitted a number of systematic reviews to be undertaken. These have reported on survival rates for single-and multiple-surface ART restorations in primary teeth, single surface restorations in permanent teeth and ART sealants^65^ and compared ART versus amalgam restorations^47^. Currently there is a paucity of data on the survival of Class II and multi-surface restorations in permanent teeth and those studies that have reported on these are either of rather short duration, or have rather small sample sizes^8^. The reason for the lack of data is most probably multifactorial, both due to the age groups commonly used for ART survival studies where caries lesions involving multi-surfaces are relatively rare, and also because access to Class II lesions in permanent teeth can be difficult with hand-instruments alone, until the lesion is large and the marginal ridge has been weakened by the caries process.

For multi-surface ART restorations in primary teeth, the systematic review of van't Hof, et al.^65^ (2006) reported that the survival rates of such restorations were low. More recent studies have confirmed this finding, although some studies^33^^,^^64^ show much lower survival rates than those reported in other studies, the reasons being far from clear.

Recommendation: Research is required to clarify the application of the ART approach for the management of multi-surface and Class II carious lesions in permanent teeth.

Recommendation: Further research is required to improve the success rate of ART restorations in multi-surface and Class II carious lesions in primary teeth.

### Research on the use of ART as a fissure sealant

ART sealants are an extension of the ART approach for non-cavitated teeth at risk of caries, where a high-viscosity restorative glass ionomer is used to seal vulnerable pits and fissures, or those with caries only involving the enamel^21^. even though an evaluation of ART sealants featured in the first field trial of ART in Thailand^24^, the systematic review of ART conducted by van't Hof, et al.^65^ (2006) reported that the number of studies investigating the retention and caries preventive effect of ART sealants was low. This continues to be the case even though results from existing studies are very encouraging^29^. Moreover, ART sealants offer several advantages over resin-based sealants in terms of the lack of need for strict moisture control and that they can easily be placed in outreach situations e.g. in school populations without recourse to dental clinic facilities. Further studies are therefore warranted.

Frencken and Holmgren^21^ (1999) consider that, when evaluating sealants, “biological outcomes should take precedence over mechanical outcomes”. In other words, since sealants are usually placed to prevent the onset or to arrest early caries lesions, the true outcome of their success should be expressed in terms of how they have managed to prevent or arrest a lesion from progressing. In a systematic review of the caries-preventive effect of resin-based and glass ionomer sealants, Beiruti, et al.^3^ (2006) concluded that there was no evidence that either resin-based or glass ionomer sealant material was superior to the other in preventing dentine lesion development in pits and fissures over time. The decision as to which material to use for sealing might therefore be dependent upon factors such as cost and clinical setting.

Recommendation: Additional long-term studies should be conducted to evaluate both mechanical and biological outcomes of ART sealants in comparison to resin-based sealants in different clinical settings, provided by different levels of oral health personnel, and in populations with different levels of caries risk.

Recommendation: Further research should be undertaken as to the value of using ART sealants to seal sound occlusal surfaces, as against sealing only those surfaces with early enamel lesions, or dentine lesions with small cavity openings e.g. <1 mm.

Recommendation: Studies should be initiated to investigate why, despite the loss of glass ionomer cement from pits and fissures sealed with ART sealants, these surfaces appear to be more caries resistent than pits and fissures previously sealed with resin-based sealants.

### Research on the success of repaired ART restorations

An important component of the Minimal Intervention (MI) approach to the management of dental caries is that restorations deemed to have failed should, where technically possible, be repaired rather than replaced in order to conserve sound tooth tissue^62^. In their book on ART, Frencken and Holmgren^21^ (1999), discuss the management of defective and failed restorations and their repair. While there have now been many studies documenting the survival of ART restorations, there are no studies on the survival of repaired or replaced ART restorations. Such information would help identify situations where a repair of an ART restoration is likely to result in long term success and where a repair should be avoided and another type of restoration might be considered.

Recommendation: Research should be initiated on the survival of repaired and replaced restorations taking into account such factors as the initial cavity size, shape and location, and the nature of the primary failure.

### Research on patient acceptance, pain and anxiety

Many publications report that subjectively ART is very well accepted by patients since no drill is used, there is almost no noise and rarely is an injection required for local anaesthesia. The few studies which have been published on the subject of patient acceptance, pain and anxiety related to ART have been reviewed by Leal, et al.^37^ (2010). In this review, it is pointed out that there is little information available regarding pain and discomfort related to the ART approach for both adults and young children. In those studies that do exist, the results are difficult to interpret because of issues concerning methodology and because confounding factors such as age, gender, operator influence and cultural aspects have not been taken into account^37^.

Recommendation: Research on dental fear, pain and anxiety relating to ART and other restorative procedures require further investigation using standard and accepted methodology taking into account possible confounding factors.

## COMMUNITY, PUBLIC HEALTH AND HEALTH SERVICES RESEARCH

### Research on the use of ART in specific population groups

In most countries the proportion of elderly people is increasing. The United Nations states that population aging is unprecedented, a global phenomenon and is having major consequences and implications on all facets of human life^63^. The aging of populations also imposes new challenges to health care systems, both in terms of the type of care required and access to care for a population which might be medically compromised and where mobility might be severely reduced. The high portability of ART offers an opportunity to care for such patients outside the traditional dental care setting.

To date only two studies have investigated the use of ART in elderly populations, one in Finland^31^ and the other in Hong Kong^39^. While both of these studies showed the value of the ART approach in such populations, both studies were of rather short duration with relatively small sample sizes. Additional studies on the use of the ART approach in the elderly are therefore required for this important and ever growing population group.

Another void in the area for ART research concerns its application for people with special needs such as those whose oral health care is compromised by physical, mental, medical or social disability. Because of the difficulties in managing these patients they tend to receive less oral health care than the general population, and when care is delivered the operator might need to resort to the use of sedation or protective stabilization^26^. Since ART is considered to be generally well accepted by patients because of the “no needle, no drill, no noise” characteristic, it might offer a viable alternative to traditional approaches. Currently only one publication on the use of ART in this field has been published^48^.

Early childhood caries (ECC) is a serious public health problem in disadvantaged communities in both developing and industrialized countries^11^. To date there is only limited evidence on the use of the ART approach in young infants^17^. Figueredo^19^ (2006) has proposed that further research should include both a quantitative and qualitative evaluation of the ART in such infants where there is not only an evaluation of the clinical performance of the ART restorations placed in children with ECC but also an investigation of the mothers' perceptions about the ART approach. To this could be added research on how well young infants tolerate the ART approach, since Ammari^2^ (2007) points out general anesthesia is often required when treating very young children, adding to morbidity and introducing the risk of mortality.

Recommendation: Research on ART should be conducted in specific population groups with the emphasis on the elderly, people with special needs and in young infants with Early Childhood Caries.

### Research on science transfer and application

The late Eva Mertz-Fairhurst in a guest editorial for the Journal of Dental Research on “Pit-and-fissure sealants: a global lack of science transfer?” quotes Genco who, on assuming the role of President of the International Association for Dental Research in 1991, stated: “The dental research community has been entrusted with enhancing the oral health of society, and with this trust comes a responsibility to transfer the fruit of our findings to society”^42^.

In this editorial Mertz-Fairhurst poses three questions relating to the use of fissure sealants for the prevention of dental caries: 1. Why is there a time lag in the adoption of pit and fissure sealants as a routine caries preventive procedure for children and teenagers? 2. Why are sealants not used by the majority of dentists, and, 3. Can anything be done by the dental research community to facilitate the utilisation of sealants by dental clinicians? In responding to these questions she cites certain barriers, such as the dental education system, attitudes and practices of the dental profession, including that sealants might pose an economic threat and finally reticence of insurance schemes to pay for the provision of sealants.

There are many parallels between the slow uptake of the use of sealants by dentists and the routine use of Atraumatic Restorative Treatment.

### Research on the teaching of ART in dental schools

A common observation amongst respondents to the internet survey was that many dental schools were slow to adopt and practice concepts of Minimal Intervention dentistry (MI), including ART, in their curricula. The reasons for this are not clear and are no doubt multifactorial. Currently there is little published information available on the adoption of MI and ART in dental curricula around the world and what barriers might exist. In preparation for the ART symposium during the 3^rd^ Latin American Regional Meeting of the IADR, in Venezuela (2009), this issue was investigated with respect to Brazilian dental schools^50^. This survey suggests that ART is taught in many of the dental schools in Brazil which is very encouraging. However these findings should not be considered to be the norm worldwide, since the ART approach continues to have a very active following in Brazil, which is not the case for many other countries.

It has been said that it is “easier to move a graveyard that to change a dental curriculum”^57^ and this epitomises the difficulties in changing curricula to adopt new concepts and approaches, difficulties which are not unique to the dental curriculum^66^. Regrettably, failure to implement teaching of evidence-based minimal intervention approaches such as ART, within a dental curriculum, not only puts dentists at a disadvantage but ultimately their patients and their communities.

Recommendation: Research should be conducted to determine the extent and nature of teaching on minimal intervention for caries and ART within dental curricula and to identify the barriers which might exist in incorporating such approaches.

### Research on the use of ART in general dental practice

A recurrent theme from many of the respondents was the need to investigate why oral health care authorities and dentists still hesitate to adopt ART as part of their treatment protocols, even though the results from clinical studies demonstrate its effectiveness for dental caries management. It is inevitable that one reason is that some dentists have neither heard of ART nor practiced it^7^, or are not trained and do not feel competent to practice it^41^. However, for those who are cognisant of the approach, it would be useful to identify whether the barriers to using ART are economic, relate to social and peer norms or relate to ingrained beliefs that ART is a substandard and temporary treatment, to be considered only for the poor and disadvantaged. An example of this latter mentality is demonstrated by a policy statement by the American Academy of Pediatric Dentistry^1^ (2008), where ART, previously renamed “Alternative Restorative Treatment” and now referred to as “Interim Restorative Treatment”, is considered a “provisional technique in conventional pediatric restorative dentistry” in “…situations in which traditional cavity preparations and/or placement of traditional dental restorations is not feasible”^1^.

Frencken and Holmgren^21^ (1999) have always stressed the need for training in ART even for existing dental practitioners since although the ART approach might look deceptively simple to the uninitiated, there are many finer details to the approach that need to be observed to ensure consistent and reliable results. As with many dental procedures, the results obtained in a clinical study, even under field conditions, might not always reflect those obtained in dayto-day dental practice, as is evident from the study of Burke, et al^7^ (2005). For that reason dental practice-based research networks have an important role to play, not only for traditional treatment, but also to evaluate new and innovative approaches such as ART^4^.

Recommendation: Research should be conducted to determine the use of ART within dental practice and possible barriers that exist to its use.

Recommendation: Research should be conducted into the effectiveness of ART provided in dental practice.

### Research on the use of ART in public oral health systems

In spite of endorsement of the ART approach by the World Health Organisation in 1994^67^, by the FDI World Dental Federation in 2002^62^, and by the Pan American Health Organisation in 2006^54^, relatively few countries have incorporated ART comprehensively into their national oral health care systems, Mexico being a notable exception^27^. Investigations have been carried out in South Africa^44^ and in Tanzania^35^ asking government dentists what they consider to be the major barriers that exist to using ART. In both these cases the barriers include: work load, lack of provision of materials and perception of clinical skill. Such research provides valuable information at the individual dentist level, but there remains no information at the health policy decision level concerning the barriers to the use of ART in public oral health systems.

Recommendation: Research should focus on the use of ART in national oral health care systems. This includes investigation of the barriers why oral health care authorities and dentists still hesitate to adopt ART.

### Research on the cost effectiveness of ART

Cost effectiveness studies of different oral health treatment approaches are rather rare in the literature, but such studies are important to any publicly funded oral health care scheme to ensure that the maximum benefit is achieved with the resources available. Such studies can be complicated and the results are not always applicable to situations outside those to where the study was conducted. For example, the cost of the treatment must take into account such factors as the cost of the oral health care provider, the equipment and materials required, the time necessary to undertake the treatment and the setting where the treatment is provided. Since these and other factors can differ between countries and regions, data from research conducted in, for instance, a Scandinavian country might not be directly applicable to a Latin American country and *vice verse*.

Some studies on the cost effectiveness of the ART approach have been conducted in South Africa^46^ and in Ecuador, Panama and Uruguay as part of the PRAT study of PAHO^54^. However, all these studies are deficient on methodological grounds.

Recommendation: Research should examine the cost effectiveness of ART against other minimally invasive approaches and traditional treatment in different settings, both for the primary and permanent dentition.

### Research on the Basic Package of Oral Care (BPOC)

The success of the ART approach in making it possible to provide restorative and preventive care in almost any setting led to the development of a Basic Package of Oral Care (BPOC), work commissioned by the WHO^22^. This model for oral care is based on self care and prevention involving toothbrushing with an effective and affordable fluoride toothpaste (AFT); Oral Urgent Treatment for the relief of pain, infection and trauma (OUT); and ART. There is a sound evidence base for all the components of the BPOC and the authors of the package have called for demonstration programs to evaluate the tenets of this model of basic oral care. While a few studies on the BPOC are in progress in a number of countries, there remains a need for further research of this and other oral health packages.

Recommendation: Demonstration programs should be established to evaluate the Basic Package of Oral Care in all its aspects including affordability, accessibility, acceptability, sustainability.

## CONCLUSIONS

Since its conception, the ART approach has consistently been the subject of research in order to place the approach within a sound evidence base for its application to improve oral health. As a result of this, the approach has evolved and improved as more was known about its strengths and weaknesses. There is now a robust, reliable and ever-growing evidence base concerning the clinical applications of the ART approach. This however should not lead to complacency amongst the research community, since the current exercise seeking opinions about future ART research has identified several further topics for research. Some of these should be considered as “nice to know” rather than “need to know”, since research outcomes are unlikely to make significant changes to the way that the ART approach is applied on a day-to-day basis. Other areas are perhaps more important, for instance to identify the barriers that prevent the utilisation of ART and other Minimal Intervention approaches in routine dental practice and public oral health systems. By identifying such barriers action can be taken to reduce or remove them. Such research will need to call on expertise outside the dental research field and involve sociologists, health economists and others to ensure that quality research is achieved.

It is hoped that the definition of a new research agenda, as detailed in this publication, will stimulate researchers in academia, public health administrators and industry to invest time and effort in this essential area of health care. It is also hoped that funding agencies will recognise the need to wholeheartedly support these activities with the objective of improving oral health, not only locally within countries, but globally.

ART has been a remarkable success story in the history of dentistry and oral health and the authors have a firm conviction that it will be possible to improve on this success through further research. In this respect, it is only fitting to conclude by quoting the words of one of the respondents to our internet survey, who wrote: “Your request for input from the clinical and research communities verifies selfless giving and collective problem solving to address needs of the underserved. I think that's what ART has been from the inception.” Such a statement makes all our efforts worthwhile.

The acquisition of knowledge is the mission of research, the transmission of knowledge is the mission of teaching and the application of knowledge is the mission of public service.

James A. Perkins (1911-1998) - Seventh president of Cornell University.
